# The Utility of Human Immune System Mice for High-Containment Viral Hemorrhagic Fever Research

**DOI:** 10.3390/vaccines8010098

**Published:** 2020-02-22

**Authors:** David M. Wozniak, Kerry J. Lavender, Joseph Prescott, Jessica R. Spengler

**Affiliations:** 1 Center for Biological Threats and Special Pathogens, Robert Koch Institute, 13353 Berlin, Germany; wozniakd@rki.de (D.M.W.); PrescottJ@rki.de (J.P.); 2 Department of Biochemistry, Microbiology and Immunology, College of Medicine, University of Saskatchewan, Saskatoon, SK S7N 5E5, Canada; kerry.lavender@usask.ca; 3 Viral Special Pathogens Branch, Division of High-Consequence Pathogens and Pathology, Centers for Disease Control and Prevention, Atlanta, GA 30329, USA

**Keywords:** humanized mice, HIS mice, NSG, TKO, BLT, filovirus, Ebola, EBOV, Marburg, Crimean-Congo Hemorrhagic fever, Rift Valley Fever, hantavirus, VHF, hemorrhagic fever virus

## Abstract

Human immune system (HIS) mice are a subset of humanized mice that are generated by xenoengraftment of human immune cells or tissues and/or their progenitors into immunodeficient mice. Viral hemorrhagic fevers (VHFs) cause severe disease in humans, typically with high case fatality rates. HIS mouse studies have been performed to investigate the pathogenesis and immune responses to VHFs that must be handled in high-containment laboratory facilities. Here, we summarize studies on filoviruses, nairoviruses, phenuiviruses, and hantaviruses, and discuss the knowledge gained from using various HIS mouse models. Furthermore, we discuss the complexities of designing and interpreting studies utilizing HIS mice while highlighting additional questions about VHFs that can still be addressed using HIS mouse models.

## 1. Introduction

Viral hemorrhagic fevers (VHFs) are a diverse group of animal and human illnesses that cause severe multisystem dysfunction syndromes, often accompanied by damage to the vascular system and associated signs of hemorrhagic disease. VHFs are a diverse group of RNA viruses including filoviruses, bunyaviruses, arenaviruses, flaviviruses, and rhabdoviruses [1]. Research of these highly pathogenic viral families is often limited to high-containment biosafety level 3 (BSL-3) or BSL-4 laboratories due to the severity of the resulting disease and lack of efficacious therapeutics or vaccines. The overall global burden of VHFs has historically been considered low compared to other diseases, such as influenza, human immunodeficiency virus, and malaria. However, it is substantial; annual cases of Lassa fever, for example, are estimated at 100,000–300,000 [2], and the 2013–2016 outbreak of Ebola virus (EBOV; species *Zaire ebolavirus*; family *Filoviridae*) in western Africa [3] along with the ongoing outbreak that began in 2018 in the Democratic Republic of Congo [4,5] highlight the potential for VHFs to cause large-scale international epidemics.

VHFs are the result of direct virus-mediated effects and, to varying degrees, of both direct and indirect effects of the immune response to infection. Therefore, investigating the immune response during infection is critical for understanding the underlying mechanisms of pathogenesis. However, patient samples are often limited in number and type (restricted to sera or peripheral blood mononuclear cell preparations), particularly for studying early stages of infection, as samples are typically obtained during disease or convalescence. Animal model development aims to characterize species that develop clinical signs and pathology that at least partially recapitulate the clinical course of disease in humans, with the hope that they also mimic the underlying mechanisms of disease. Importantly, animal models can serve as a tool to comprehensively examine immune responses over the course of virus infection. 

Several mouse models have been developed to study VHFs, most of which rely on serially passaging virus to generate a disease-inducing variant or on the use of immunodeficient mouse strains. While there are advantages to studies using mice, the utility of the data generated from these models for understanding human disease is not always clear. To improve the translational quality of mouse models and to facilitate immunological and disease investigations, mice can be engrafted with human cells using a variety of approaches. However, the use of humanized mouse models brings unique disadvantages along with utility. Here, we provide an overview of recent use of human immune system (HIS) mice to study high-containment VHFs, with a focus on filoviruses, nairoviruses, phenuiviruses, and hantaviruses. Herein, we review the work to date involving HIS mice and the knowledge garnered from these models. We then address the complexities of designing and interpreting HIS mouse studies and discuss additional knowledge that can still be gained.

## 2. Background

### 2.1. Humanized Mice

The term “humanized mice” encompasses a wide range of mouse models that have been manipulated to express human genes, or to support engraftment or transplantation of human cells and/or tissues. “HIS mice” are a subset of humanized mice that are generated by xeno-engrafting human immune cells or tissues and/or their progenitors into immunodeficient mice, and contain viable components of a human immune system. Both the background mouse strain and the humanization procedure contribute to the resulting level of human immune reconstitution and immune system functionality. Overall, HIS models continue to improve as background strains become more immunocompromised and as knowledge of the factors that allow for robust humanization within the xenogeneic murine environment increase. 

### 2.2. Generation of HIS Mice

A variety of background mouse strains are used to generate HIS mice, but all background strains need to be immunodeficient in order to prevent rejection of the human xenograft by the murine immune system. The earliest models took advantage of the severe combined immunodeficiency (SCID) (*Pkrdc*^scid^) strain devoid of B and T cells that would otherwise mediate rejection of the human graft [6]. Recombination-activating gene knock-out (*Rag*^−/−^) strains are also useful B and T cell-deficient HIS hosts [7,8]. Back-crossing SCID mice to the NOD strain [9] and deleting the common gamma chain (*Il2r*ɣ*_c_*^−/−^) [10] on both NOD/SCID [11] and *Rag*^−/−^ [12] backgrounds further improved human graft acceptance, primarily by eliminating natural killer (NK) cell-mediated graft rejection. Additionally, the NOD allele of the inhibitory SIRP*α* receptor recognizes CD47 on human cells, thereby preventing phagocytosis of the xenograft by the murine myeloid compartment in NOD/SCID mice [13]. Myeloid-mediated graft rejection in other HIS strains has been surmounted either by knocking in the human *Sirpα* gene [14] or by knocking out the murine *Cd47* gene [15], resulting in a human xenograft-tolerant phagocytic compartment. With each improvement in overall immunosuppression and tolerance within the murine hosts, human immune engraftment and reconstitution levels have increased both in cell number and repertoire in HIS models.

While preventing anti-graft immune reactions in the host mouse strain is key to successful reconstitution, additional factors are necessary for optimal development and functionality of the engrafted human immune system. Thus, efforts have been made to remedy these deficiencies by introducing human transgenes into immunodeficient background strains. Mice transgenic for human cytokine genes have improved development of cell lineages reliant on cytokines that are not cross-reactive between mice and humans. For example, mice transgenic for human GM-CSF, IL-3, and SCF support greater development of human myeloid lineages [16], whereas mice transgenic for human IL-2 [17] and IL-15 [18] support better NK cell development and survival. Functionality, as opposed to levels of reconstitution, has also been improved through transgenic expression of human leukocyte antigens (HLA) in graft recipient mouse strains. Mice transgenic for class I and/or class II HLA [19,20] have T cells educated in the context of human HLA and thus are able to mediate antigen-specific human HLA-restricted T cell responses toward both the HLA-matched human xenograft and the HLA-expressing murine parenchyma. 

The methods used to implant human immune cells and/or tissues into the mouse recipient also impact the level of reconstitution, functionality, and longevity of the human graft. Here we describe three common methods to produce HIS mice. The first method involves injecting peripheral blood leukocytes (PBL) from a human donor directly into an adult mouse [21]. The resultant PBL-mice mainly harbor human T cells [22] and are particularly useful HIS models for vaccine studies [23,24]. However, since the immune cells do not develop within the mouse, they are highly xenoreactive, and the mice develop graft versus host disease (GVHD) within months [25]. A second method of humanization involves transferring CD34^+^ hematopoietic stem cells (HSC) into host animals that have undergone myeloablative conditioning to facilitate implantation and long-term hematopoiesis by the graft. HSCs are sourced from fetal liver, cord blood, bone marrow, or induced in peripheral blood, and can be implanted into either newborn or adult recipient mice to produce humanized mice (hu-mice) [26]. Both B and T cells develop within the murine environment, so reconstitution can be sustained for approximately a year with little or no incidence of GVHD. However, immune functionality is not ideal in hu-mice, at least partly due to the lack of HLA-mediated T cell selection in the murine thymus, which since has been improved by using HLA transgenic strains [19,20]. Lastly, the bone marrow, liver, thymus (BLT) method of humanization also involves transplanting CD34^+^ HSCs into preconditioned mice. The HSCs in this model are generated from fetal liver, and small pieces of autologous fetal liver and thymus are additionally implanted under the kidney capsule of the mouse [27]. The tissue implant develops into a surrogate human thymus for the maturation and selection of highly functional, HLA-restricted T cells. Unlike other methods, BLT humanization results in sustained and high-level multi-lineage reconstitution, mucosal immunity, antigen-specific B and T cell responses, organized lymphoid tissues, and antibody class-switching [15]. Thus, BLT-mice are the most highly reconstituted and functional HIS model produced to date. Nevertheless, the BLT model also has limitations, including an underdeveloped B cell compartment that diminishes over time [28] and the development of lethal xenogeneic GVHD [29] in some mouse strains. To date, a variety of HIS models has been used to study VHFs, representing each of these humanization approaches (Table 1).

## 3. Use of HIS Mice in VHF Research

Research involving high-consequence VHFs, including studying immune responses to infection, has long been hampered by the limitations of small animal models. Wild-type laboratory mouse strains, for example, are often resistant to disease with non-adapted viruses, or are overly sensitive to infection, as in the case of Rift Valley fever virus (RVFV) [32]. Mice with defective immune systems like SCID and *Rag*^−/−^ strains, on the other hand, allow for productive replication of viruses in vivo but with limited pathology. Some disease signs can be recapitulated in mice with defects in the antiviral type I interferon response (*Stat1*^−/−^ or *Ifnar*^−/−^ strains), supporting in vivo studies of therapeutics [33,34] and immune modulators [35]. However, the underlying disease mechanisms in these models might not truly reflect human disease, and the immune systems of these animals are highly divergent from those of humans, thus greatly limiting the translational quality of the research.

**Figure 1:**
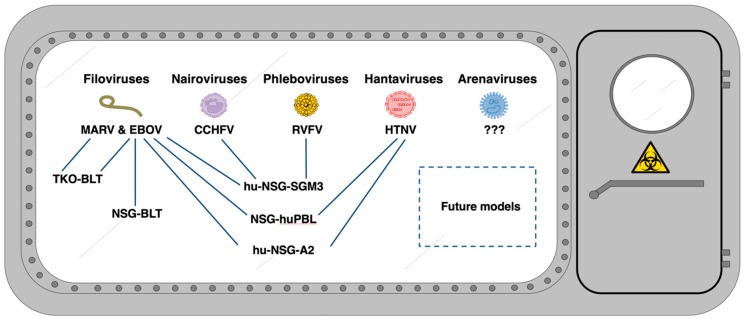
HIS models used in research of high-containment VHFs.

HIS mouse models are beginning to address a knowledge gap in VHF research, enabling investigations of human immune cell reactions in vivo during productive infection. While research using HIS mice has increased, relatively few VHF studies have been published to date, and these are limited to only a subset of the known high-containment viruses (Figure 1). However, these studies provide a foundation for advancing VHF work and have provided interesting and novel findings that are reviewed below and summarized in Table 2.

### 3.1. Filoviruses

Filoviruses like Marburg virus (MARV) and ebolaviruses have been identified as the cause of small human VHF outbreaks since the 1960s and more recently have been the etiologic agents of large-scale outbreaks with high case fatality rates [3,45]. MARV was the first filovirus discovered in 1967 [46,47,48], following importation to Marburg, Germany, via infected grivets sourced from Uganda for the development of a poliomyelitis vaccine. The first ebolavirus species, *Zaire ebolavirus* (EBOV), was subsequently discovered in 1976 [49,50,51] near the Ebola River in what is now the Democratic of Republic of Congo, and has since posed a major threat to communities in central and western Africa. Currently, six species of ebolaviruses have been described, including EBOV, *Sudan ebolavirus* (SUDV), *Tai Forest ebolavirus* (TAFV), *Bundibugyo ebolavirus* (BDBV), *Reston ebolavirus* (RESTV), and *Bombali ebolavirus* (BOMV). In vitro studies using human monocyte-derived dendritic cells and macrophages have provided strong evidence of the immunotropic nature of filoviruses [52,53,54], which was supported in early non-human primate (NHP) studies [55]. Given the central role of immune cell populations in infection and disease, HIS mouse models are particularly interesting for filovirus research by providing a unique opportunity to study human immune cell interactions and responses in vivo.

#### 3.1.1. Ebolaviruses

Early reports of ebolavirus studies in HIS mice were based on work in NOD/SCID *Il2rɣ_c_*^−/−^ mice engrafted with human PBL (NSG-huPBL) using both mouse-adapted and wild-type EBOV [36]. Similar to infection in non-HIS mice, wild-type EBOV infection did not result in lethal disease in NSG-huPBL. Later, studies were performed using more advanced HIS models [56,57]. The human cell-engrafted NOD.Cg-*Prkdc^scid^ Il2rg^tm1Wjl^* Tg (HLA-A2.1) 1Enge/Sz mice (hu-NSG-A2) that express human HLA-A2 provided the first platform for a lethal EBOV mouse model without the need of prior virus adaption to the host [38]. Lethal disease is observed in hu-NSG-A2 following intraperitoneal [58] or intranasal inoculation with the prototypic EBOV Mayinga strain [37]. 

EBOV isolated from the more recent West Africa outbreak (Makona strain) can also induce a lethal outcome in hu-NSG-A2 mice, but only to a limited extent [37]. When other species of ebolaviruses were evaluated in hu-NSG-A2 mice, SUDV, RESTV, TAFV, and BDBV all caused lower lethality rates than EBOV [37], reflecting relative case-fatality rates reported for the respective species in human cases. However, while only non-pathogenic human RESTV infections have been recorded [59], RESTV caused a 20% lethality rate in hu-NSG-A2 mice, a rate previously exceeded only in infected NHPs [60,61]. Lethality in RESTV-infected hu-NSG-A2 mice also differed from the outcome noted in another humanized mouse model, humanized NOD.*Cg-Prkdc^scid^ Il2rg^tm1Wjl^* Tg (CMV-IL3, CSF2, KITLG) 1Eav/Mloy SzJ (hu-NSG-SGM3) mice. Hu-NSG-SGM3 mice, which are transgenic for human IL-3, CSF-2, and SCF to support improved reconstitution of the human myeloid compartment [16], display no disease signs of RESTV infection, even though they provide a disease model for EBOV infection [42]. In EBOV-infected hu-NSG-SGM3 mice, murine-origin hepatocytes stained positive for EBOV antigen, and liver damage (histopathology, liver enzyme abnormalities) recapitulated that seen in human disease. In contrast, RESTV infection was mostly restricted to liver macrophages without evidence of hepatic disease. Both EBOV and RESTV replicated to similar titers in hu-NSG-SGM3 mice except in the liver, in which RESTV replicated less efficiently. Although lethality differed in hu-NSG-A2 and hu-NSG-SGM3 mice, notably, both studies indicated an association between viral load in liver and outcome of ebolavirus infection.

In support of EBOV research in HIS mice, studies in BLT humanized NOD/SCID *Il2rɣ_c_*^−/−^ mice (NSG-BLT) have demonstrated parallels in immune responses to infection reported in humans and in HIS mice. A massive, non-specific release of host cytokines, or “cytokine storm”, [62,63] is one aspect of EBOV pathogenesis implicated in severe disease. Cytokine profiles during EBOV infection of NSG-BLT mice [40] recapitulated those in fatal human Ebola virus disease (EVD), including high expression of M-CSF, IL-6, IL-8, and TNFα, and the chemokines fractalkin, MIP-1β, and MCP-1 [62,64,65]. Interestingly, the data suggested human donor-specific variations on the outcome of the disease [40], which should be considered in conjunction with the possible effects of engraftment efficiency, as was shown in hu-NSG-A2 mice [38]. In addition, flow cytometry analysis of human cells in EBOV-infected BLT humanized C57BL/6 *Rag2*^−/−^ *Il2rɣ_c_*^−/−^ *Cd47*^−/−^ (TKO-BLT) mice [41], ≈60% of which succumbed to infection by 22 days post-infection (dpi), suggested that EBOV infection induced myeloid cell dysfunction and skewing of macrophage subsets in vivo at late time points, in line with prior in vitro experiments using human cells [54,55].

#### 3.1.2. MARV

Studies of MARV infection in HIS mice are much more limited than ebolavirus studies. Only one report, using the TKO-BLT model, investigated MARV infection in HIS mice [41]. In humans, MARV disease is comparable in severity to EBOV disease. However, in TKO-BLT mice, MARV infection resulted in limited disease compared to EBOV infection; its cellular tropism was more prolific in murine hepatocytes, with lower levels of infection of Kupffer cells than observed during EBOV infection. In contrast to the skewed and dysfunctional myeloid cell phenotypes observed during EBOV infection, flow cytometric analysis of human cells from MARV-infected animals suggested a more functional and balanced immune response to infection. 

### 3.2. Nairoviruses

Ticks of the genus *Hyalomma* are the main vector Crimean-Congo hemorrhagic fever virus (CCHFV; family *Nairoviridae*, genus *Orthonairovirus*) throughout regions of Mediterranean Europe, Balkans, Middle East, Asia, and Africa. Livestock are prone to infection but remain asymptomatic while still being important for transmitting the virus to humans. In humans, symptoms range from subclinical infection to hemorrhagic syndromes, with fatal outcomes in 5–30% of cases [66]. Susceptibility to disease from CCHFV in mice has been limited to strains deficient in type I interferon responses [67,68]. These mice show a very rapid and highly lethal disease progression [69,70], which is likely largely due to their genetically defective antiviral response rather than a faithful recapitulation of human Crimean-Congo hemorrhagic fever (CCHF)-pathogenesis [70]. 

To date, there is only one report on CCHFV in HIS mice. Hu-NSG-SGM3 mice were inoculated intraperitoneally with one of two virus strains: infection with CCHFV-Turkey was uniformly lethal, while CCHFV-Oman-infected mice all survived. Reports of human CCHF case fatality rates suggest that CCHFV is less pathogenic in Turkey than in Oman, but this may be due in part to under-reporting of mild cases in Oman [71]. Importantly, while the difference in outcome is notable, these studies were initiated to evaluate outcome in the model and not designed as a direct comparison of strain pathogenicity. Overall, disease progression in hu-NSG-SGM3 mice was slower than in IFN-deficient models, and thus more comparable to human disease. Within inflammatory foci, CCHFV immune reactivity was mostly localized in endothelial cells and immune cells like histiocytes, multinucleated giant macrophages, and Kupffer cells [43], but it was unclear whether immune cell targets were of human or murine origin. The main histological finding in lethally CCHFV-Turkey-infected hu-NSG-SGM3 mice was evidence of a neurotropic infection, resulting in meningitis, infection of astrocytes and glial cells, and infiltration of immune cells into the brain [43]. Neurological signs in human CCHF have been reported, but are uncommon. The relevance of these findings to human disease remains unclear, as does the mechanism by which the presence of human immune cells may have conferred susceptibility to infection. Investigating CCHFV in HIS mice remains very preliminary. While fatal disease in hu-NSG-SGM3 mice is a promising outcome, additional studies using these and other HIS mice are needed, focusing on the role of the immune response in disease. Such studies will aid in determining the utility of HIS mice in CCHF research.

### 3.3. Phenuivirus

RVFV (family *Phenuiviridae*, genus *Phlebovirus*) is a mosquito-borne VHF transmitted by six genera of mosquitos, including *Aedes* and *Culex* [72], in Sub-Saharan Africa, Egypt, the Arabian Peninsula, and Madagascar. Modeling Rift Valley fever (RVF) disease in mice has been challenging due to the sensitivity of these animals to infection; immunocompetent mice uniformly succumb to disease in 2–3 days. This rapid and severe clinical course does not reflect the human phenotype and suggests that underlying mechanisms of human disease are not fully recapitulated. RVFV can strongly inhibit the type I interferon response [73], which is a critical factor in disease progression in mice. Artificially attenuating the virus by deleting RVFV’s type I interferon-inhibiting NSs gene alleviates the disease in type I interferon-competent mice [73]. Studies in HIS mice investigated whether the presence of human immune cells could dampen the clinical course to more accurately model human disease. However, intramuscularly infected hu-NSG-SGM3 mice succumbed to disease at similarly fast rates [44]. Human cytokine expression in the hu-NSG-SGM3 model in part mirrored cytokines seen in fatal patient outcomes, but increased expression of murine cytokines was also observed during infection. This highlights the importance of considering the remaining immune function of the murine background of HIS mice when evaluating the role of immune responses in disease progression.

### 3.4. Hantaviruses

Hantaviruses are harbored by rodents, shrews, and bats, but only rodent-borne hantaviruses have been reported to cause hemorrhagic syndromes in humans [74]. Hantavirus research using HIS mice is limited. The only study to date compared human HSC-engrafted NSG mice (hu-NSG) with hu-NSG-A2 mice in regard to possible cytotoxic T lymphocyte (CTL)-induced immunopathologies during infection with Hantaan virus (HTNV; family *Hantaviridae*, genus *Orthohantavirus*) [45]. HTNV-induced disease progression was accelerated in the HLA-A2-expressing hu-NSG-A2 mice compared to hu-NGS mice that did not express transgenic human MHC I, despite similar viral replication in both models. Endothelial cells are considered the primary target cells of hantaviruses [75]. Expression of human HLA-A2 on murine endothelia in the hu-HLA-A2 mouse would allow them to be targeted by CTLs, possibly contributing to the accelerated pathology observed in this model. HTNV infection caused a reduction in human platelet counts in hu-NSG-A2 mice while murine platelets remained unaffected. While a clear explanation for this phenomenon is missing, elimination of virally infected human megakaryocytes or platelets in HLA-A2-expressing mice or differing human and murine β3 integrin interactions with hantaviruses on activated platelets could be responsible [45].

## 4. What We Have Learned about Using HIS Mice for VHF Studies

Overall, we have learned that HIS mice do not completely model human disease, even though VHF infection may result in clinical disease and pathology. Nonetheless, they serve as useful tools for studying specific events in virus infection. VHF studies to date have demonstrated that HIS models only occasionally recapitulate a subset of features of human disease. This is not unexpected, as the human immune features of each specific HIS model influence the disease process. For example, CTL-mediated pathogenesis has been observed in the hu-NSG-A2 model of EBOV [37] resulting in liver pathology. This is due to the expression of HLA-A2-restricted epitopes that can be presented by infected murine cells and targeted by human CTLs. However, CD4^+^ T cell responses and several other compartments of the immune system are deficient, limiting modeling of all immune aspects that contribute to this and other features of disease. Similarly, hepatic pathology has been observed in the hu-NSG-SGM3 model of EBOV [39], but likely occurred through an alternative mechanism with myeloid cell involvement. Therefore, although hepatocytes are infected in both models during EBOV infection, independent mechanisms contribute to liver disease in each one.

HIS mouse studies to date have shown value in investigating individual immune pathways, but have not provided a definitive answer regarding the most important events in human disease, nor a complete system to examine interrelated events. These events include dendritic cell and macrophage infection; immune target cell dysregulation; antigen presentation to CD4^+^ T cells; involvement of B cells and antibody responses; infection of parenchymal cells; and killing of infected cells by CTL or NK cells. The interactions that can be investigated vary dramatically depending on the mouse model. For example, adding thymus xenografts to the HIS model allows T cells to be educated on the human thymus and to recognize antigens presented by human antigen presenting cells. On the other hand, CD8^+^ T cells only incidentally interact with murine MHC I but provide a more complete picture of the early events in which dendritic cells, macrophages, and monocytes are infected or take up antigen and present antigen to T cells and B cells, thereby activating these cells. While not an accurate model of VHF disease course from beginning to end, HIS mice provide a more faithful human immune system to examine specific features in a hypothesis-driven manner. For example, by comparing various viruses using the BLT system we have found that differences in dendritic cell and T cell responses exist and can be characterized even between closely related viruses [41]. 

## 5. Limitations of HIS mice

Introducing human cells into mice using any of the approaches described to date does not result in the same proportions of immune cells as seen in humans. Transgenic expression of human cytokines in HIS mice [16], as well as the use of BLT xenografts, has drastically improved these models [15]. Furthermore, regardless of the model, generation of HIS mice results in experimental subjects that often differ in their immune system composition, introducing complex variability into experiments. Donor material can also vary from batch to batch and from institution to institution. The presence of these confounding variables is challenging for study design and data interpretation, requiring more test subjects. Despite these challenges, the possibility of studying immune systems of differing human genetic backgrounds is also a great advantage of humanized mouse models. Another powerful feature of HIS mice is that multiple genetically identical animals can be produced from the human-sourced tissue, providing a means for experimentation using multiple “clones” of a single human immune system. 

HIS mouse model limitations vary, specifically when considering which aspects of the immune response can be investigated in disease. For example, although human immune cells can react natively to viruses surveyed from their surroundings or introduced through infection of the immune cells, human CD8^+^ T cell interaction with murine parenchymal cells is severely limited in most HIS models. Without the expression of human MHC I on mouse cells, an important part of the antiviral immune response by CD8^+^ T cells is absent. Non-hematopoietic cells in these mouse models are often antigen-positive for virus. Especially common is the infection of endothelial cells [41,42,43] as well as infection of hepatocytes and Kupffer cells [41,76,77]. CD8^+^ T cell responses are thought to be important in the development of disease caused by VHFs [78,79,80]. Transgenic expression of human MHC I molecules in HIS mice [38,45] was attempted to address this problem, but this approach restricts the CD8 T cell responses to only HLA-A2-compatible viral epitopes and T cell receptors, even though many other immune-dominant epitopes exist for other MHC I alleles (e.g., A3 and B7) [81].

Other limitations are more universal to current HIS mouse models, including the inability to research the influence of any antibody isotypes besides IgM on viral infection; this is due to severely limited class switching from IgM to other isotypes in most humanized mouse models [82]. The effect of neutralizing antibodies on disease outcome is significant in VHFs like EVD [83,84]. However, non-neutralizing antibodies can also fulfill important roles in opsonization, activation of complement [81] and antibody-dependent cellular cytotoxicity (ADCC) [85] during disease. While neither phagocytosis nor ADCC are effectively mediated by antibodies of the IgM isotype, IgM does effectively activate complement. Complement activation is impaired in NSG mice due to a C5 deficiency [86] but HIS models based on the C57BL/6 [15] or other complement-competent background strains could be used to study this effector mechanism. In vivo mouse studies of complement-dependent tumor depletion by the humanized Rituximab antibody [87] suggest that human antibodies would be able to activate mouse complement in HIS models.

Other factors that create challenges in HIS mouse studies are the dynamic relationships between human and mouse cells and their respective immune responses. In HIS mice, much of the murine immune system is absent or non-functional due to genetic modification or irradiation, limiting the influence of the murine immune response. However, residual immune cells and other cells that produce cytokines and innate immune mediators, such as type I interferons are present in HIS mice. This response likely influences not only the outcome of infection, but also the responses of the human immune cells. While murine responses to VHF infection in HIS mice have been described [44], studies to date have not specifically addressed how the background murine response might influence the activities of the human immune system in these mice.

In HIS mice, there is a disconnect between the cytokine response of the human immune system, and both the remaining mouse myeloid system and the non-hematopoietic mouse cells. While immune cells are often considered the main producers of important cytokines, other cells can also strongly impact the cytokine milieu [88]. Thus, non-hematopoietic murine endothelia and hepatocytes could contribute significantly to cytokine production, along with other murine myeloid cells that many VHFs preferentially infect. Limited cross-reactivity of human and mouse cytokines [89] prevents a full assessment of causes and effects in these models, as does omitting or neglecting assessment of mouse cytokines during these studies. Unobserved parallel murine cytokine messaging from endothelial or other murine cells harboring virus could occur in HIS mouse disease models [41,42,43], but is not commonly investigated. Even less is known about the extent of human immune cell interactions with murine tissue ligands. Tissue ligands are another important factor of communication to immune cells, yet by not studying cross-reactive effects between mice and humans, the immune cells are essentially kept in undefined stimulatory milieus. Finally, VHFs also target non-immune cells during infection [41,42,43], which further complicates studies in HIS mice due to the limited interaction of human immune cells with murine soma. In the absence of key murine immune cells, viral infection of murine cells cannot be ordinarily antagonized, which may additionally alter recapitulation of immunological events found in natural infection.

## 6. Conclusions

VHF research in humanized mouse models remains in its infancy, as we continue to learn about the limitations and utility of these models. Although generating HIS mouse models and interpreting data from such studies may be more complex than using other mouse models, HIS mice remain a valuable tool to model HIS responses and interactions in vivo; in particular for analyzing organ-specific immune reactions and infection patterns. For example, while using rodent-adapted virus in mice may generate uniform lethality, these models may not be as informative for human disease when examining the interaction between the virus and various components of the immune system. In addition, aspects of human disease progression may be reflected better in HIS mice. For example, CCHFV studies are restricted to type I interferon-deficient mice, in which infection progresses too rapidly for sufficient investigation of the immune responses [67,68,69,70]. In HIS mice, CCHF progression was delayed compared to *Ifnar^−/−^* models, offering novel opportunities for immunological investigations.

Many questions remain about VHF diseases that may be addressed in HIS mice. The factors that differentiate why some people develop subclinical infection while others develop severe or fatal disease are unknown, for example. Using different donors for humanized mouse generation could be helpful in revealing whether underlying donor differences in the immune system could be responsible for such strongly divergent outcomes of infection. Additionally, some VHFs have yet to be investigated in HIS mice, including, notably, arenaviruses. The family *Arenaviridae* includes many hemorrhagic fever-causing species [90] that are predominantly spread by small rodents, but few aspects of human immune responses to these viruses are known. For example, most immunological knowledge of humans with Lassa fever, the most characterized arenaviral disease, is based on a few case studies of single patients [91], single cases evacuated to first-world countries [92,93], and serological studies [94], leaving large sets of immunological analyses still unexplored. NHPs [95,96,97,98] and small animal models like guinea pigs [99,100,101] and type I interferon-deficient mouse strains (*Ifnar^−/−^* and *Stat1*^−/−^) [33,79,102,103] are commonly used to study these diseases. Even though human immune cells are considered an early target of arenaviruses, no studies in humanized mice have yet been published [104,105].

New HIS mouse models are being developed that may advance our knowledge of VHFs. Numerous methods are being introduced to express human cytokines in HIS models such as transgenic expression, injection of recombinant proteins or plasmid DNA, or use of adenovirus- associated viruses to express proteins of interest. Additionally, mouse backgrounds expressing additional HLA class I alleles, including HLA-A2, A11, A24, B7, B27, and Cw3, and class II alleles like HLA-DR1, DR2, DR3, DR4, and DQ8 [106], either alone or in combination, are currently in development. Efforts are also underway to find ways to enhance the function of the universally underdeveloped and short-lived B cell compartment, including transgenic expression of human MHC II [107], transgenic expression of human IL-6 [108], and knockout of the *Cmah* gene in the murine background [109]. Knocking out murine MHC has also been shown to reduce incidence of GVHD in susceptible strains [110]. 

Current HIS models all have potential benefits and drawbacks that need to be considered during experimental design. Thus, choosing the most appropriate HIS model for any experiment requires careful reflection of whether the hypothesis can be tested based on the specific functional components of the model and the interplay between immune components of both murine and human origin. Ultimately, new HIS models should aim to more fully recapitulate all the interconnected facets of immunity. We envision a time when HIS models are combined to take advantage of key modifications to enhance all aspects of immunity, like, for example, BLT humanization of an immunodeficient strain that exhibits GVHD resistance and expresses full graft-matched HLA class I and II haplotypes and a suite of essential human cytokines.

The initially intended application of HIS mice to VHF research was two-fold: to establish a small animal model that more faithfully recapitulated human disease, and to advance investigations into the role of the immune system in infection and disease. We have learned that engraftment of human immune cells and tissues is not sufficient to completely mirror human disease and that the lack of a direct interaction between human immune cells and the murine parenchyma may be the primary limitation in modeling pathology. Still, much has been learned and remains to be investigated regarding the involvement of the immune response in VHF disease, components of which can be studied using HIS mice. Currently, no vaccine evaluations in HIS mice for the viruses discussed here have been reported. However, given the infancy of the field, continuing to understand the models, particularly for VHFs, is necessary for designing and interpreting any putative vaccine work in the future. Studies to date have highlighted the challenges and complexities of HIS mouse studies. Nonetheless, the use of HIS mice in VHF research has been informative. Current and newly developed HIS models will continue to enable the study of complex immune interactions that occur in vivo upon infection and provide valuable insight into the human immune response to VHFs.

## Figures and Tables

**Table 1:** Human immune system (HIS) mice used in viral hemorrhagic fever research.

Common Name	Background Strain	Transgenic Human Proteins	Humanization Method	Notes	Ref.
NSG-huPBL	NOD/SCID *Il2rɣ_c_*^−/−^	None	PBL	Highly functional T cell engraftment; GVHD within ≈4–5 weeks	[11,23]
hu-NSG-A2	NOD/SCID *Il2rɣ_c_*^−/−^	HLA-A2	HSC	HLA-matched human T cells can recognize infected mouse cells	[19]
NSG-BLT	NOD/SCID *Il2rɣ_c_*^−/−^	None	BLT	Susceptible to GVHD	[27]
hu‑NSG‑SGM3	NOD/SCID *Il2rɣ_c_*^−/−^	SCF GM-CSF IL-3	HSC	Improved human myeloid cell development	[30]
TKO-BLT	C57BL/6 *Rag2*^−/−^*Il2rɣ_c_*^−/−^*Cd47*^−/−^	None	BLT	GVHD resistant	[15,31]

PBL, peripheral blood lymphocytes; BLT, bone marrow-liver-thymus; GVHD, graft vs. host disease; HSC, hematopoietic stem cells.

**Table 2:** Summary of viral hemorrhagic fever (VHF) research in HIS mice.

Agent	Mouse Model	Virus Strain	Route, Dose	Survival	Main Findings	Ref.
***Filoviridae***						
EBOV	NSG-huPBL	MA-EBOV Mayinga	IP, 10³ PFU IP, 10³ PFU	0% 100%	MA-EBOV infections induced human lymphocyte apoptosis and human cytokine response, but wild-type EBOV (Mayinga) infection did not	[36]
	hu-NSG-A2	Mayinga Mayinga Makona	IP, 10³ FFU IN, 10³ FFU IN, 10³ FFU	13% (1/8) 7% (1/14) 57% (4/7)	Disease severity may correlate with human cell engraftment efficiency	[37,38]
	hu‑NSG‑SGM3	Makona	IM, 10³ FFU	50% (3/6) ^‡^	Higher EBOV replication in liver compared to RESTV investigated in parallel	[39]
	NSG-BLT	Mayinga Makona	IP, 10², 10³ and 10^5^ TCID_50_ IP, 10² TCID_50_	0% (0/13) 25% (1/4)	Donor-dependent variations in severity observed	[40]
	TKO-BLT	Makona	IM, 10^3^ FFU	44% (4/9) *	Accumulation of dysfunctional M2-like macrophages	[41]
SUDV	hu-NSG-A2	Gulu-808892	IN, 10^3^ FFU	29% (2/7)	Lethality rate analogous to human case fatality rates	[37]
TAFV	hu-NSG-A2	Pauleoula-CI	IN, 10^3^ FFU	82% (9/11)	Lethal TAFV infection produced high serum AST but low viremia	[37]
BDBV	hu-NSG-A2	Bundibugyo-200706291	IN, 10^3^ FFU	29% (2/7)	Lethality rate similar to human case fatality rates	[37]
RESTV	hu‑NSG‑SGM3	RESTV-Pennsylvania	IM, 10^3^ FFU	100% (6/6) ^‡^	No gross pathology, RESTV levels lower than EBOV levels in liver	[39]
	hu-NSG-A2	RESTV-Pennsylvania	IN, 10^3^ FFU	80% (12/15)	Lethal RESTV infection correlated with inflammation and high RESTV replication in the liver	[37]
MARV	TKO-BLT	Angola-368	IM, 10^3^ FFU	25% (2/8) *	MARV replicated similarly to EBOV, but induced more functional innate immune response	[41]
***Nairoviridae***						
CCHFV	hu‑NSG‑SGM3	Turkey-200406546 Oman-199809166	IP, 10^4^ TCID_50_ IP, 10^4^ TCID_50_	0% (0/5) 100% (6/6)	Strain-dependent severity. Time to death 13–23 days. Animals with terminal outcomes showed higher levels of perforin positive CD8 T cells. CCHFV-Turkey associated hepatic and neurological histopathology	[42]
***Phenuiviridae***						
RVFV	hu‑NSG‑SGM3	rZH-501 rZH-501	IM, 10^1^ TCID_50_ IM, 10^4^ TCID_50_	0% (0/7) 0% (0/7)	Human immune cells did not alter disease course; murine cytokines still influential in humanized mouse models	[43]
***Hantaviridae***						
HTNV	hu-NSG-A2 hu-NSG	Strain 76-118 Strain 76-118	IP, 10^5^ FFU IP, 10^5^ FFU	25% (2/8) * 25% (2/8) *	Human immune cells increase pathology; human CD8 T cell responses accelerated early pathology; human platelet loss during infection	[44]

^‡^, until 14 dpi; *, until 21 dpi; IM, intramuscular; IN, intranasal; IP, intraperitoneal; MA, mouse-adapted.
